# The Clinical and Biological Effects of Receptor Expression-Enhancing Protein 6 in Tongue Squamous Cell Carcinoma

**DOI:** 10.3390/biomedicines11051270

**Published:** 2023-04-25

**Authors:** Chung-Chih Tseng, Chung-Ching Hung, Chih-Wen Shu, Cheng-Hsin Lee, Chun-Feng Chen, Mei-Shu Kuo, Yu-Ying Kao, Chun-Lin Chen, Luo-Ping Ger, Pei-Feng Liu

**Affiliations:** 1 Institute of Medical Science and Technology, National Sun Yat-sen University, Kaohsiung 80424, Taiwan; caviton@gmail.com; 2 Department of Dentistry, Zuoying Branch of Kaohsiung Armed Forces General Hospital, Kaohsiung 81342, Taiwan; 3 Department of Otolaryngology, Zuoying Branch of Kaohsiung Armed Forces General Hospital, Kaohsiung 81342, Taiwan; happyforever1010@gmail.com; 4 Institute of BioPharmaceutical Sciences, National Sun Yat-sen University, Kaohsiung 80424, Taiwan; cwshu@g-mail.nsysu.edu.tw; 5 Department of Biomedical Science and Environmental Biology, College of Life Science, Kaohsiung Medical University, Kaohsiung 80708, Taiwan; r980084@kmu.edu.tw; 6 Department of Stomatology, Kaohsiung Veterans General Hospital, Kaohsiung 81362, Taiwan; 261463@gmail.com; 7 Department of Biotechnology, Chia Nan University, Tainan 71710, Taiwan; mashu0417@hotmail.com (M.-S.K.); viviakao7175@gmail.com (Y.-Y.K.); 8 Department of Biological Sciences, National Sun Yat-sen University, Kaohsiung 80424, Taiwan; chunlinchen@mail.nsysu.edu.tw; 9 Department of Medical Education and Research, Kaohsiung Veterans General Hospital, Kaohsiung 81362, Taiwan; lpger0329@gmail.com; 10 Department of Medical Research, Kaohsiung Medical University Hospital, Kaohsiung 80708, Taiwan; 11 Center for Cancer Research, Kaohsiung Medical University, Kaohsiung 80708, Taiwan; 12 Institute of Biomedical Sciences, National Sun Yat-sen University, Kaohsiung 80424, Taiwan

**Keywords:** tongue squamous cell carcinoma, receptor expression-enhancing protein 6, malignancy, prognosis

## Abstract

There are currently no effective biomarkers for the diagnosis and treatment of tongue squamous cell carcinoma (TSCC), which causes a poor 5-year overall survival rate. Thus, it is crucial to identify more effective diagnostic/prognostic biomarkers and therapeutic targets for TSCC patients. The receptor expression-enhancing protein 6 (REEP6), a transmembrane endoplasmic reticulum resident protein, controls the expression or transport of a subset of proteins or receptors. Although it was reported that REEP6 plays a role in lung and colon cancers, its clinical impact and biological role in TSCC are still unknown. The present study aimed to identify a novel effective biomarker and therapeutic target for TSCC patients. Expression levels of REEP6 in specimens from TSCC patients were determined with immunohistochemistry. Gene knockdown was used to evaluate the effects of REEP6 in cancer malignancy (colony/tumorsphere formation, cell cycle regulation, migration, drug resistance and cancer stemness) of TSCC cells. The clinical impact of REEP6 expression and gene co-expression on prognosis were analyzed in oral cancer patients including TSCC patients from The Cancer Genome Atlas database. Tumor tissues had higher levels of REEP6 compared to normal tissues in TSCC patients. Higher REEP6 expression was related to shorter disease-free survival (DFS) in oral cancer patients with poorly differentiated tumor cells. REEP6-knocked-down TSCC cells showed diminished colony/tumorsphere formation, and they also caused G1 arrest and decreased migration, drug resistance and cancer stemness. A high co-expression of REEP6/epithelial–mesenchymal transition or cancer stemness markers also resulted in poor DFS in oral cancer patients. Thus, REEP6 is involved in the malignancy of TSCC and might serve as a potential diagnostic/prognostic biomarker and therapeutic target for TSCC patients.

## 1. Introduction

Tongue squamous cell carcinoma (TSCC) is one of the major subsites of oral squamous cell carcinoma (OSCC) and a tumor with poor prognosis [1]. The incidence of TSCC is increasing around the world. The current therapeutics for TSCC are surgery, chemotherapy and radiotherapy. Regardless of advances in diagnosis and therapy, the 5-year overall survival rate is less than 50% for TSCC patients [2]. Thus, the need for new biomarkers and therapeutic targets for TSCC patients is urgent.

Receptor expression-enhancing proteins (REEPs) belong to the DP1/Yop1p family. At least six REEP family members (REEP1–6) have been identified [3] and divided into two subfamilies (REEP1–4 and REEP5–6) according to their structural and sequence homology [4]. REEPs act as membrane-shaping adapter proteins in endoplasmic reticulum (ER) to regulate the expression or transport of cargo proteins from ER to the Golgi complex or plasma membrane [5]. For example, it controls the membrane expression and trafficking of a subset of G protein-coupled receptors (GPCRs) which is implicated in various types of cancers [6,7]. However, the role of REEP6 in cancers, especially in TSCC, is still unclear.

This study compares REEP6 expression between normal and tumor tissues and investigates the association of REEP6 expression with clinicopathological outcomes in TSCC patients. We also analyze the biological role of REEP6 in TSCC cells, including cellular growth/migration and drug resistance/cancer stemness. Our findings demonstrate the clinical impact and biological role of REEP6 in TSCC patients.

## 2. Materials and Methods

### 2.1. Specimens

This study collected paraffin-embedded tissues from 250 TSCC patients with informed consent at Veterans General Hospital of Kaohsiung, Taiwan. All patient specimens were collected from the Department of Pathology at KVGH between 1993 and 2006, and their records were archived in a consistent process. Normal uvula from 35 patients with sleep apnea were also collected for paraffin-embedded tissues. The study was evaluated and approved by the IRB of Kaohsiung Veterans General Hospital (IRB: VGHKS11-CT12-13, approval date on 4 February 2015) in accordance with the Declaration of Helsinki. Transcriptome profiling of 303 oral cancer patients including most of the TSCC patients in The Cancer Genome Atlas (TCGA) database (https://cancergenome.nih.gov (accessed on 25 February 2021) were downloaded for survival analysis.

### 2.2. Immunohistochemistry (IHC)

Tissue microarray paraffin blocks were cut at a thickness of 4 μm for IHC analysis [8]. Sodium citrate buffer (10 mM, pH 9.0) was used for antigen retrieval for 10 min at 125 °C and 3% H_2_O_2_ in methanol was used for blocking endogenous peroxidase activity for 30 min. REEP6 antibody (diluted 1:100, Abcam, Trumpington, Cambridge, UK) was incubated with tissue sections at 4 °C for 16 h. The Novocastra Novo-Link Max Polymer Detection System was used for color development.

### 2.3. IHC Scoring

Semi-quantitative cytoplasmic staining was performed for quantification of REEP6 levels. The intensity score (0/negative, 1/weak, 2/moderate, 3/strong) and percent score (0 (<5%), 1 (5–25%), 2 (26–50%), 3: (51–75%), 4 (>75%)) for positive cells were added to produce a final score. A detailed description of IHC scoring is provided in our previous study [9].

### 2.4. Cell Culture

The TSCC cells (SAS cells) were cultured in Dulbecco’s Modified Eagle Medium (Gibco, Invitrogen Corporation, Carlsbad, CA, USA) containing 10% FBS, 100 µg/mL streptomycin, 100 U/mL penicillin, and 1% L-glutamine and then maintained within 5% CO_2_ at 37 °C.

### 2.5. Transient Transfection

Non-targeting siRNA or siRNA against REEP6 (10 nM, S41034, Ambion, Austin, TX, USA) were mixed with RNAiMAX reagent and then transfected into 2 × 10^5^ cells/well in 6 well for 72 h (Life Technologies, Carlsbad, CA, USA) [10].

### 2.6. Real-Time Quantitative PCR (RT-qPCR)

The TRIzol reagent (Invitrogen Life Technologies, Carlsbad, CA, USA) was used for RNA isolation. The cDNA synthesis was performed using SuperScript II RNase H-Reverse Transcriptase (Invitrogen Life Technologies, Carlsbad, CA, USA). The levels of mRNA were analyzed with the SYBR Green Master Mix (Applied Biosystems, Foster City, CA, USA). The REEP6 primer (Forward: 5′-TGTGTGGCTCACCTACTGG-3′; Reverse: 5′-CGCGCTGATACAGCATGAGA-3′) was used in this study. The gene expression was calculated according to cycle threshold (CT) normalized to the internal control gene (GAPDH) and the 2(-Delta Delta C(T)) method was used to analyze the relative changes in gene expression. The RT-qPCR efficiency of each group was presented in percentage.

### 2.7. Western Blotting

The total proteins of cells were separated by the SDS-PAGE electrophoresis and transferred onto a nitrocellulose membrane, which was then blocked with 5% skim milk for 1 h at room temperature and incubated with the REEP6 antibody (diluted 1:2000, Abcam) at 4 °C overnight. Afterwards, the nitrocellulose membrane was incubated with the HRP-labeled secondary antibody at room temperature for 1 h, and the ECL reagent was used for chemiluminescence imaging (GeneGnome XRQ, SYNGENE, Cambridge, UK).

### 2.8. Colony Formation

About 1 × 10^3^ cells were cultured in 6-well plate, and the media were refreshed every three days until colonies formed. Colonies were then fixed with 3.75% paraformaldehyde (*v*/*v*) and stained with 0.25% crystal violet (*w*/*v*). The stained colonies were quantified using ImageJ software.

### 2.9. Volume and Cell Viability of Spheroids

The silenced cells were seeded at 5 × 10^3^ cells/mL in a 96-well ultra-low attachment microplate (Corning Costar, Cambridge, MA, USA) following the formation of spheroids for 3 days. Afterwards, the spheroids were treated with/without anti-cancer drugs including cisplatin (CIS, 100 μM, Sigma-Aldrich Corporation, St. Louis, MO, USA) and paclitaxel (PTX, 0.2 μM, Selleckchem, Houston, TX, USA) for 24 h. The spheroid volume was calculated by V = 4/3 πR^3^, and the cell viability of spheroids treated with cancer drugs was observed with the CellTiterGlo 3D assay (Promega, Madison, WI, USA) [10].

### 2.10. Cell Cycle Analysis

After being fixed with ice-cold 75% ethanol, the cells were stained with propidium iodide (50 µg/mL, Sigma-Aldrich, St. Louis, MO, USA) for 30 min. Subsequently, the percentage of cells in each cell cycle was analyzed with the FACScan analyzer (Becton, Dickinson and Company, Franklin Lakes, NJ, USA) and quantified using FlowJo software (Tree Star, Ashland, OR, USA).

### 2.11. Migration

About 2 × 10^5^ cells were seeded in IBIDI Culture-Inserts (IBIDI, Inc., Planegg, Germany), which were located in 12 well plates, for 24 h. Next, the inserts were removed, and the wound-healing activity was observed for 4 h until the migration distance was close to that in the control cells.

### 2.12. Statistical Analysis

SPSS software (version 20.0, IBM-SPSS Inc., Chicago, IL, USA) was utilized for clinical data analysis. The comparison of REEP6 expression between normal and tumor tissues of TSCC patients was estimated using the Mann–Whitney U test. Overall survival (OS) and disease-free survival (DFS) were defined according to the TCGA database. The associations of REEP6 expression and the co-expression level of the two genes with DFS were analyzed using univariate Cox’s regression or multivariate Cox’s regression adjusted for group comparison or for different clinicopathological outcomes. The receiver operating characteristic curve was used to dichotomize ‘high’ and ‘low’ gene expression. The interaction of the REEP6 protein and its associated protein was predicted using the Search Tool for Retrieval of Interacting Genes/Proteins (STRING) database (https://string-db.org/ (accessed on 30 April 2021).

## 3. Results

### 3.1. The Clinical Impact of REEP6 in TSCC Patients

**Figure 1:**
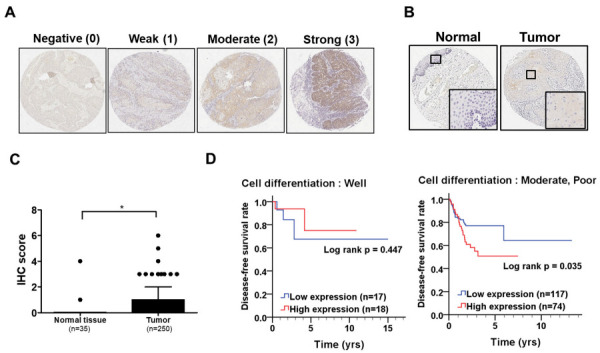
REEP6 expression in oral cancer patients. (**A**) Intensity of REEP6 staining was scored into 0/negative, 1/weak, 2/moderate, and 3/strong to quantity REEP6 protein levels in the tumor tissues of TSCC patients. (**B**) Levels of cytoplasmic REEP6 were compared between normal and tumor tissues in TSCC patients. (**C**) IHC scores for REEP6 expression were measured in 35 normal tissues and 250 tumor tissues from TSCC patients. The significant difference was indicated as * *p* < 0.05. (**D**) DFS levels in oral cancer patients stratified by cell differentiation were compared depending on levels of REEP6.

To assess the clinical significance of REEP6 in TSCC, we initially evaluated the expression levels of the REEP6 protein between normal and tumor tissues of TSCC patients by IHC using a numerical scale for scoring intensity (Figure 1A). It showed that the protein levels of REEP6 were significantly higher than those of normal uvula tissues (Figure 1B–C; *p* = 0.012, Table 1). Moreover, we used another cohort of oral cancer patient from the TCGA database to investigate the association of REEP6 expression with the prognosis of patients. We found that the high level of REEP6 was not related to OS (Table S1) but related to poor DFS in oral cancer patients having poor cell differentiation (*p* = 0.035, Figure 1D; crude hazard ratios = 1.80, 95% confidence interval (CI) = 1.04–3.14, *p* = 0.038, Table 2). This indicates that high REEP6 expression is associated with tumorigenesis and shorter DFS in TSCC patients given certain clinicopathological outcomes.

### 3.2. The Role of REEP6 in the Growth of TSCC Cells

**Figure 2:**
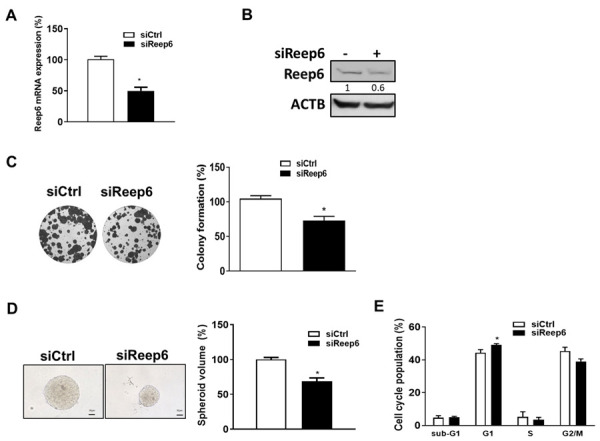
Cell viability and cell cycle progression of REEP6-silenced SAS cells. The mRNA and protein levels of REEP6 in SAS cells were evaluated by (**A**) RT-qPCR and (**B**) Western blotting to determine silencing efficiency, respectively. (**C**) Colony formation of REEP6-knocked-down SAS cells was monitored and quantified. (**D**) Spheroid volume of REEP6-knocked-down SAS cells was measured. (**E**) Cell cycle distributions of REEP6-knocked-down SAS cells were analyzed by flow cytometry. The 10 nM scrambled siRNA (siCtrl) or siRNA against REEP6 (siREEP6) were transfected into SAS cells for 72 h. All data were represented as the average ± SD from 3 independent experiments. The significant differences between the scrambled control and knocked-down cells were indicated as * *p* < 0.05. The full-length blot is presented in Figure S1.

To further examine the biological role of REEP6 in TSCC, REEP6 was knocked down for 72 h in SAS cells, and the silencing efficiency was examined by RT-qPCR (Figure 2A) and Western blotting (Figure 2B). We found that the colony formation (Figure 2C) and tumorsphere formation (Figure 2D) were both decreased in REEP6-knocked-down SAS cells compared to the levels of control cells. Cell cycle analysis also indicated that REEP6-knocked-down cells exhibited an arrested G1 phase (Figure 2E), suggesting that REEP6 participates in TSCC cell growth by regulating the cell cycle progression.

### 3.3. The Role of REEP6 in Migration of TSCC Cells

**Figure 3:**
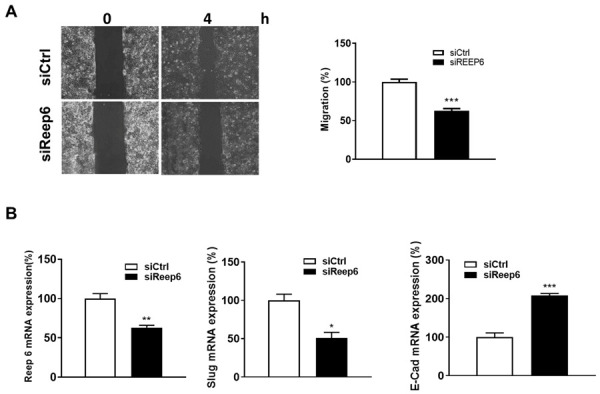
Cell migration of REEP6-knocked-down SAS cells. (**A**) The effect of REEP6 on cancer cell mobility was measured by the wound-healing assay. (**B**) Expression of EMT markers (Slug and E-cad) in REEP6-knocked-down SAS cells were measured. The 10 nM scrambled siRNA (siCtrl) or siRNA against REEP6 (siREEP6) were transfected into SAS cells for 72 h. All data were represented as the average ± SD from 3 independent experiments. The significant differences between the scrambled control and knocked-down cells were indicated as * *p* < 0.05, ** *p* < 0.01, and *** *p* < 0.001.

We next investigated if REEP6 plays the role in the metastasis of TSCC. In the results, REEP6-knocked-down SAS cells showed decreased cell migration in comparison to control cells (Figure 3A). The gene level of two epithelial–mesenchymal transition (EMT) markers was also verified in REEP6-knocked-down SAS cells. The results showed the level of Slug was decreased but the level of E-cadherin was increased in REEP6-knocked-down SAS cells (Figure 3B). Moreover, we found that oral cancer patients with a high co-expression of REEP6/Slug had poor DFS (adjusted hazard ratio (AHR) = 2.69, 95% CI = 1.04–6.95, *p* = 0.041, Table 3), indicating that REEP6 might be involved in TSCC metastasis.

### 3.4. The Role of REEP6 in the Drug-Resistant Cancer Stemness of TSCC Cells

**Figure 4:**
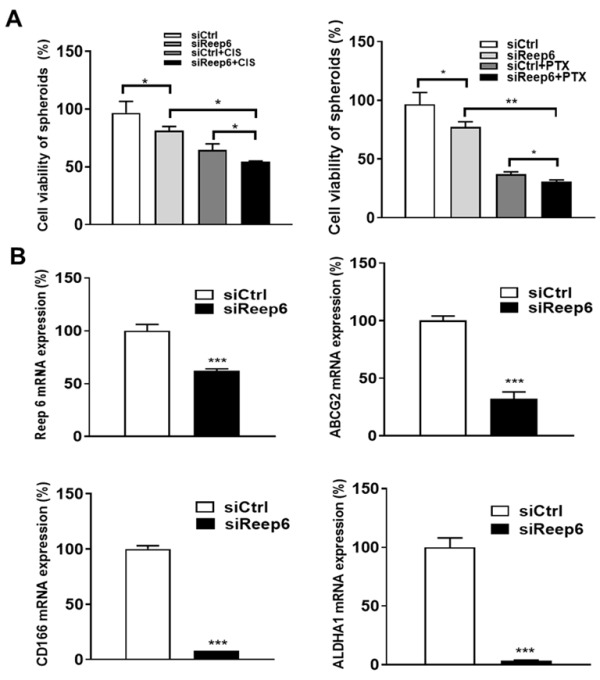
Cancer stemness of REEP6-knocked-down SAS cells. (**A**) The cell viability of spheroids from SAS cells silenced with scrambled siRNA or siRNA against REEP6 for 3 days was detected in the absence or presence of cisplatin (CIS, 100 μM) or paclitaxel (PTX, 0.2 μM) for 24 h. (**B**) mRNA levels of cancer stemness markers in REEP6-knocked-down SAS cells were assessed by RT-qPCR. The 10 nM scrambled siRNA (siCtrl) or siRNA against REEP6 (siREEP6) were transfected into SAS cells for 72 h. All data were represented as the average ± SD from 3 independent experiments. The significant differences between scramble control and knocked-down cells were indicated as * *p* < 0.05, ** *p* < 0.01, and *** *p* < 0.001.

We have shown that the high expression level of REEP6 is positively correlated with poor DFS in oral cancer patients, implying that REEP6 plays a role in drug resistance. To determine whether REEP6 is also involved in drug resistance, the sensitivity of scrambled or REEP6-knocked-down SAS cells to cisplatin (CIS) and paclitaxel (PTX) in spheroids was compared. The results show that REEP6-silenced cells exhibited decreased cell viability and increased sensitivity to CIS and PTX compared to the scrambled cells (Figure 4A). We further measured the expression levels of several cancer stemness markers in the scrambled or REEP6-knocked-down SAS cells and found that expression levels of the ATP-binding cassette transporter G2 (ABCG2), cluster of differentiation 166 (CD166) and aldehyde dehydrogenase A1 (ALDH1A1) were significantly decreased in REEP6-knocked-down SAS (Figure 4B). Moreover, a high co-expression of REEP6/CD166 (AHR = 2.28, 95%CI = 1.10–4.75, *p* = 0.027, Table 4), REEP6/ABCG2 (AHR = 3.65, 95%CI = 1.42–9.41, *p* = 0.007, Table 4) and REEP6/ALDH1A1 (AHR = 3.25, 95%CI = 1.34–7.90, *p* = 0.009, Table 4) was related to the DFS of oral cancer patients in the TCGA database. These data suggest that REEP6 is involved in drug-resistant cancer stemness for TSCC.

## 4. Discussion

REEP6 is expressed in most tissues, including membranes of the retina, liver and testis [11,12]. It is an ER resident adaptor protein [13] and plays a role in the expression and transport of intracellular proteins/receptors, such as GPCR [5]. It correctly sorts certain proteins via clathrin-coated vesicles to the selected membrane sites [14]. Moreover, REEP6 expression is regulated by the bZIP transcription factor NRL in rod photoreceptors [11]. REEP6 mutations cause autosomal-recessive retinitis pigmentosa [15,16], and three novel REEP6 variants have been found among ethnic Chinese [17]. In addition, REEP6 with a novel nonsense variant was found to be associated with a sporadic rod-cone dystrophy case [18]. Interestingly, REEP6 gene therapy could rescue retinal degeneration in REEP6 mutant mice [19]. REEP6 also regulates adrenergic signal transduction in adipocytes, and its inactivation causes obesity-related metabolic dysfunction [6]. A few studies have examined the potential roles of REEP6 in various types of cancer. For example, REEP6 polymorphisms at positions 992 and 996 have been associated with colon cancer [20], and low levels of REEP6 are significantly correlated with reduced cell growth and invasion in lung cancer cells [21]. However, its role in TSCC has not been studied. The study finds that (i) expression levels of REEP6 are increased in tumor tissues of TSCC patients compared to that in normal tissues; (ii) high expression levels of REEP6 are related to the poor DFS of oral cancer patients with poorly differentiated tumors; (iii) REEP6 contributes to the growth, migration, drug resistance and cancer stemness in TSCC cells; (iv) a high co-expression of REEP6/EMT marker (Slug) or REEP6/stemness markers (CD166, ABCG2, ALDHA1) is related to poor DFS in oral cancer patients.

There are six REEP family members (REEP1–6) in humans with individual functions. REEP1 mutations are associated with hereditary spastic paraplegias (HSP) due to its lack of cytoplasmic region interaction with microtubules for the ER network [22], for which the HSP was found in the Chinese population [23]. REEP1 was also found to be a biomarker of OSCC metastasis [24]. REEP2 promotes the clustering of taste receptors in lipid rafts to enhance their functions in mouse taste cells [25]. Moreover, REEP2 is a chemosensitivity-related gene in gastric cancer cells [26]. REEP3 is a positional candidate gene for autism [27]. Surprisingly, circFAT1 stimulates the progression of hepatocellular carcinoma by sponging miR-30a-5p for regulating REEP3 expression [28]. REEP4 displays direct phosphorylation-dependent interactions with 14-3-3, which regulates various tumors, metabolic diseases and neurodegenerative diseases [29]. REEP4 is also involved in maintaining the nervous system and the musculature [30]. REEP5 is a sarcoendoplasmic reticulum membrane protein in cardiac myocytes for stabilizing the highly differentiated sarcoplasmic reticulum network by generating high membrane curvature [31]. REEP6 is critical for visual signal transduction by regulating photoreceptor guanylate cyclases [6], which are associated with increased intracellular cGMP levels and the phosphorylation of protein kinase G for its downstream molecules and are involved in cell proliferation or apoptosis [32,33]. Our present study did show that silencing REEP6 inhibits cell cycle progression and sensitivity to drug in TSCC cells.

REEP6 enhances the expression and trafficking of GPCRs, which are important tumor cell surface signaling receptors [5]. Interestingly, REEP5 and REEP6 activate interleukin-8 (IL-8)/CXC chemokine receptor 1 (CXCR1, a G-protein coupled receptor) signaling to promote tumor cell proliferation/migration, angiogenesis, and macrophages/neutrophils recruitment in the tumor microenvironment in lung cancer [21]. Our current study shows the clinical impact and biological roles of REEP6 in TSCC. First, we found significantly higher expression levels of REEP6 in tumor tissues of TSCC patients, and REEP6 promotes the cell growth of TSCC cells, implying that REEP6 might be involved in the tumorigenesis of TSCC. It is known that REEP6 enhances the activation of IL-8/CXCR1 [21] and IL-8/CXCR1 via the nucleotide binding oligomerization domain containing 1 (NOD1)/receptor-interacting-serine/threonine-protein kinase 2 (RIP2) signaling pathway, which is important for head and neck squamous cell carcinoma cell proliferation [34]. It suggests that REEP6 mediated IL-8/CXCR1 via the NOD1/RIP2 signaling pathway, which might be also involved in OSCC cell proliferation. Second, REEP6 contributes to the migration of TSCC cells, and oral cancer patients with an elevated co-expression of REEP6/Slug also had poor DFS. However, our results showed that the co-expression of REEP6/Slug but no other EMT markers such as E-cadherin, N-cadherin, Vimentin, Snail and Twist1 was associated with poor prognosis of TSCC patients (Table S2). Moreover, another CXC subfamily receptor, CXCR4, promotes the invasion of OSCC via the ERK signaling pathway [35]. It implied that REEP6 might regulate Slug expression via CXCR4/ERK signaling. Interestingly, Slug can reversely elevate CXCR4 expression in human prostate cancer [36]. Thus, it will be worthwhile to determine if REEP6 is involved in the regulation of the CXCR4/Slug feedback loop for OSCC metastasis. Third, REEP6 promotes drug resistance in TSCC cells, and oral cancer patients with an elevated co-expression of cancer stemness markers (CD166, ABCG2, ALDHA1) also had poor DFS. Previous studies showed that the IL-8/CXCR1 axis could increase properties of cancer stemness [37], indicating that the involvement of REEP6 in the drug resistance of TSCC might be associated with the IL-8/CXCR1 axis.

Our results showed that the protein levels of REEP6 were significantly higher than those of normal uvula tissues. We also compared the REEP6 expression between tumor adjacent normal and tumor tissues in TSCC patients (Table S3). However, their expression levels were not significantly different. We found that the REEP6 expression was significantly increased in tumor tissues compared with that in normal tissues. Moreover, REEP6 expression in tumor adjacent normal tissues of TSCC patients was significantly higher than that in normal tissues. These results indicated that REEP6 began to express in transformed cells, but its expression level was not significantly increased in tumor tissues, implying that REEP6 might derive the tumorigenesis of TSCC.

Our results showed that only 2–3% of the G1 phase was arrested, and there is no changed sub-G1 phase in REEP6-silenced cells compared to scramble cells (Figure 2E). It is shown that REEP6 controls the membrane expression and trafficking of a subset of G protein-coupled receptors, which play a variety of physiological processes such as cell cycle regulation, apoptosis, autophagy and cellular senescence [38]. Thus, REEP6 had no effects on cell death and might play a minor role in the cell cycle progression of TSCC cells. The possibility of GBP6 being involved in other processes such as autophagy and cellular senescence for cell growth will need to be further investigated.

It is shown that the 3D sphere culture may enrich the cancer stem cell population, which is major cause of drug resistance [39]. Thus, we analyzed the effect of REEP6 on drug resistance using a 3D spheroid model and found that the combination with siREEP6 and chemotherapeutic drugs, including CIS and PTX, significantly enhanced the cytotoxic effects of drugs in spheroids. Moreover, we analyzed the effects of REEP6 on drug resistance using monolayer cultures, we found that the cell viability between scramble and siREEP6 monolayer 2D cultures (treated CIS and PAX) showed a more significant difference (Figure S2) compared to that in 3D culture. These results indicated that 3D cultures were more resistant to cancer drugs. Overall, our data indicate that the combination of cancer drugs (CIS or PTX) and siREEP6 could be applied for overcoming the multidrug resistance in TSCC.

To further investigate more mechanisms of REEP6 for its biological roles, we also predicted the interaction network of REEP6 using the Search Tool for the Retrieval of Interacting Genes/Proteins (STRING) database, and we found that REEP6 was associated with Atlastin GTPase 2 (ATL2), ATL3, killer cell lectin-like receptor F1 [40], nucleoredoxin-like 2 [41], reticulon 1–4 [42,43,44], sorting nexin 15 [45], transmembrane protein 33 (TMEM33) [46] and lipopolysaccharide induced TNF factor (LITAF) [47,48] (Figure S3), and these partners of REEP6 were reported to be associated with cancer progression. For example, REEP6 might regulate the expression and transport of TMEM33 to promote the cell proliferation of TSCC [46]. Moreover, REEP6 might also regulate LITAF for inducing cancer growth/invasion [47] or switching tumor associated-inflammation [48]. According to the prediction, further studies would be needed to investigate whether REEP6 regulates the malignancy of TSCC through the predicted interaction network.

We have indicated that REEP6 might serve as an effective diagnostic or prognostic biomarker for TSCC patients, but there are possible limitations in the study as outlined below. (1) The specificity of commercial polyclonal REEP6 antibody is not high enough, since only a small fraction of REEP6 in IHC staining and Western blot was detected. According to our data, we probably could demonstrate the relative protein levels of REEP6 between normal and tumor tissues in TSCC patients. Unfortunately, REEP6 protein expression was not associated with clinicopathological outcomes in TSCC patients (Table S4), which might be due to the limited specificity of the REEP6 antibody. Thus, we focused on analyzing the TCGA database for the REEP6 gene expression and its association with prognosis in oral cancer patients in the study. (2) Although we have used the higher invasive and metastatic potential TSCC cell (SAS) [49] to study the biological roles of REEP6, other TSCC cells with different migratory/invasive potential will also need to be evaluated. (3) According to the analyzed data from the TCGA database, we found that OSCC patients with higher REEP6 expression had poor DFS, implying that REEP6 might play a role in drug resistance [50]. However, there is no information about chemotherapy treatment such as the duration and protocol of treatment and types of chemotherapy drugs for OSCC patients from the TCGA database. Other cohorts with standard treatment guidelines will be required. Nevertheless, our study showed that REEP6 might regulate cell proliferation and drug resistance along with the expression of EMT and cancer stemness markers in TSCC cells. A high co-expression of REEP6 and these markers had unfavorable DFS in TSCC.

## 5. Conclusions

Our study indicates that REEP6 is involved in the cancer malignancy of TSCC and has potential value as a diagnostic/prognostic biomarker and therapeutic target for TSCC patients.

## Figures and Tables

**Table 1:** Comparison in REEP6 expression between normal and tumor tissues of TSCC patients.

Variables	Normal		Tumor		Z	*p*-Value *
	Mean ± SD	Median	Mean ± SD	Median		
REEP6	(n = 35)		(n = 250)			
	0.14 ± 0.69	0.00	0.45 ± 0.93	0.00	2.506	0.012

SD: Standard Deviation. *p* value *: assessed by Mann-Whitney U test. Z: z score/standard value.

**Table 2:** The association of REEP6 expression with DFS of oral cancer patients in TCGA database.

Variable	Reep6	No. (%)	CHR (95% CI)	*p* Value *	AHR (95% CI)	*p* Value ^†^
Sex						
Female	Low	44 (60.3)	1.00		1.00	
	High	29 (39.7)	1.47 (0.52−4.21)	0.470	1.57 (0.55−4.55)	0.402 ^a^
Male	Low	90 (58.8)	1.00		1.00	
	High	63 (41.2)	1.58 (0.86−2.92)	0.143	1.61 (0.87−2.99)	0.131 ^a^
Age, yrs						
≦60	Low	54 (57.4)	1.00		1.00	
	High	40 (42.6)	1.03 (0.43−2.48)	0.951	1.01 (0.42−2.44)	0.979 ^a^
>60	Low	80 (60.6)	1.00		1.00	
	High	52 (39.4)	1.97 (1.01−3.84)	0.047	1.98 (1.00−3.88)	0.049 ^a^
Cell differentiation						
Well	Low	17 (48.6)	1.00		1.00	
	High	18 (51.4)	0.50 (0.08−3.08)	0.455	0.66 (0.11−4.04)	0.650 ^b^
Moderate, poor	Low	117 (61.3)	1.00		1.00	
	High	74 (38.7)	1.80 (1.04−3.14)	0.038	1.74 (1.00−3.04)	0.051 ^b^
AJCC pathological stage						
I, II	Low	33 (63.5)	1.00		1.00	
	High	19 (36.5)	1.69 (0.42−6.75)	0.460	2.29 (0.57−9.18)	0.241 ^c^
III, IV	Low	101 (58.0)	1.00		1.00	
	High	73 (42.0)	1.52 (0.86−2.70)	0.151	1.54 (0.87−2.73)	0.140 ^c^
T classification						
T1, T2	Low	65 (67.7)	1.00		1.00	
	High	31 (32.3)	1.18 (0.46−3.04)	0.735	1.43 (0.56−3.70)	0.457 ^d^
T3, T4	Low	69 (53.1)	1.00		1.00	
	High	61 (46.9)	1.70 (0.89−3.27)	0.109	1.72 (0.89−3.29)	0.105 ^d^
N classification						
N0	Low	59 (55.1)	1.00		1.00	
	High	48 (44.9)	1.78 (0.78−4.08)	0.170	1.80 (0.77−4.19)	0.177 ^e^
N1, N2	Low	75 (63.0)	1.00		1.00	
	High	44 (37.0)	1.41 (0.70−2.82)	0.334	1.36 (0.68−2.74)	0.390 ^e^
Postoperative RT						
No	Low	53 (60.2)	1.00		1.00	
	High	35 (39.8)	1.54 (0.58−4.12)	0.387	1.70 (0.63−4.61)	0.295 ^a^
Yes	Low	75 (58.6)	1.00		1.00	
	High	53 (41.4)	1.69 (0.88−3.25)	0.117	1.72 (0.89−3.33)	0.104 ^a^

DFS: Disease-Free Survival; AJCC: American Joint Committee on Cancer, RT: Radiotherapy, CHR: Crude Hazard Ratio, AHR: Adjusted Hazard. Ratio, CI: Confidence Interval. *p* values *: measured by univariate Cox’s regression; p values: p values ^†^: measured by multivariate Cox’s regression. *p* values ^a^: adjusted for cell differentiation (well vs. moderate, poor) and AJCC pathological stage (I, II vs. III, IV). *p* values ^b^: adjusted for AJCC pathological stage (I, II vs. III, IV). *p* values ^c^: adjusted for cell differentiation (moderate, poor vs. well). *p* values ^d^: adjusted for cell differentiation (well vs. moderate, poor) and N classification (N0 vs. N1, N2). *p* values ^e^: adjusted for cell differentiation (well vs. moderate, poor) and T classification (T1, T2 vs. T3, T4).

**Table 3:** The co-expression of REEP6/Slug in DFS of oral cancer patients in TCGA database.

Variable		No. (%)	CHR (95% CI)	*p* Value *	AHR (95% CI)	*p* Value ^†^
REEP6	Low	134 (59.3)	1.00		1.00	
	High	92 (40.7)	1.56 (0.92−2.64)	0.101	1.60 (0.94−2.71)	0.085 ^a^
Slug	Low	88 (38.9)	1.00		1.00	
	High	138 (61.1)	1.35 (0.77−2.36)	0.292	1.31 (0.75−2.29)	0.343 ^a^
REEP6 (L)/Slug (L)		45 (19.9)	1.00		1.00	
REEP6 (H)/Slug (L)		43 (19.0)	1.31 (0.70−2.44)	0.398	2.54 (0.97−6.69)	0.059 ^b^
REEP6 (L)/Slug (H)		89 (39.4)	1.03 (0.60−1.77)	0.927	2.09 (0.84−5.19)	0.111 ^b^
REEP6 (H)/Slug (H)		49 (21.7)	1.42 (0.79−2.58)	0.244	2.69 (1.04−6.95)	0.041 ^b^

DFS: Disease-Free Survival; H: High expression, L: Low expression, CHR: Crude Hazard Ratio, AHR: Adjusted Hazard Ratio, CI: Confidence Interval. *p* values *: measured by univariate Cox’s regression; *p* values ^†^: measured by multivariate Cox’s regression. *p* values ^a^: adjusted for cell differentiation (well vs. moderate, poor) and AJCC pathological stage (I, II vs. III, IV) by multivariate Cox’s regression. *p* values ^b^: adjusted for group comparison by multivariate Cox’s regression.

**Table 4:** The co-expression of REEP6/cancer stemness markers in DFS of oral cancer patients in TCGA database.

Variable		No. (%)	CHR (95% CI)	*p* Value *	AHR (95% CI)	*p* Value ^†^
REEP6	Low	134 (59.3)	1.00		1.00	
	High	92 (40.7)	1.56 (0.92−2.64)	0.101	1.60 (0.94−2.71)	0.085 ^a^
CD166	Low	148 (65.5)	1.00		1.00	
	High	78 (34.5)	1.45 (0.85−2.47)	0.173	1.27 (0.74−2.18)	0.386 ^a^
ABCG2	Low	105 (46.5)	1.00		1.00	
	High	121 (53.5)	1.79 (1.03−3.13)	0.040	1.73 (0.99−3.02)	0.054 ^a^
ALDH1A1	Low	212 (93.8)	1.00		1.00	
	High	14 (6.2)	1.95 (0.83−4.56)	0.123	1.74 (0.74−4.07)	0.205 ^a^
REEP6 (L)/CD166 (L)		87 (38.5)	1.00		1.00	
REEP6 (H)/CD166 (L)		61 (27.0)	1.06 (0.59−1.93)	0.839	1.41 (0.70−2.85)	0.343 ^b^
REEP6 (L)/CD166 (H)		47 (20.8)	0.95 (0.49−1.83)	0.869	1.29 (0.60−2.77)	0.523 ^b^
REEP6 (H)/CD166 (H)		31 (13.7)	1.91 (1.03−3.57)	0.041	2.28 (1.10−4.75)	0.027 ^b^
REEP6 (L)/ABCG2 (L)		57 (25.2)	1.00		1.00	
REEP6 (H)/ABCG2 (L)		48 (21.2)	1.15 (0.62−2.14)	0.659	2.80 (1.06−7.37)	0.037 ^b^
REEP6 (L)/ABCG2 (H)		77 (34.1)	1.28 (0.74−2.20)	0.379	2.94 (1.19−7.30)	0.020 ^b^
REEP6 (H)/ABCG2 (H)		44 (19.5)	1.62 (0.90−2.94)	0.111	3.65 (1.42−9.41)	0.007 ^b^
REEP6(L)/ALDH1A1 (L)		130 (57.5)	1.00		1.00	
REEP6(H)/ALDH1A1 (L)		82 (36.3)	1.19 (0.69−2.03)	0.539	1.31 (0.75−2.31)	0.343 ^b^
REEP6(L)/ALDH1A1 (H)		4 (1.8)	0.05 (0.00−252.94)	0.488	0.00 (0.00−7.94 × 10^212^)	0.966 ^b^
REEP6(H)/ALDH1A1 (H)		10 (4.4)	2.97 (1.27−6.95)	0.012	3.25 (1.34-7.90)	0.009 ^b^

DFS: Disease-Free Survival; H: High expression, L: Low expression, CHR: Crude Hazard Ratio, AHR: Adjusted Hazard Ratio, CI: Confidence Interval. *p* values *: measured by univariate Cox’s regression; *p* values ^†^: measured by multivariate Cox’s regression. *p* values ^a^: adjusted for cell differentiation (well vs. moderate, poor) and AJCC pathological stage (I, II vs. III, IV) by multivariate Cox’s regression. *p* values ^b^: adjusted for group comparison by multivariate Cox’s regression.

## Data Availability

Not applicable.

## Supplementary Materials

The following supporting information can be downloaded at: https://www.mdpi.com/article/10.3390/biomedicines11051270/s1, Table S1: The association of REEP6 expression with overall survival of oral cancer patients in TCGA database. Table S2: The co-expression of REEP6/EMT markers in DFS of oral cancer patients in TCGA database. Table S3: The comparison of REEP6 expression between normal, tumor adjacent normal and tumor tissues of TSCC patients. Table S4: The association of REEP6 expression with clinicopathologic outcomes in TSCC patients. Figure S1: The full-length blot. Figure S2: The cell viability of REEP6-silenced SAS monolayer cultures in the presence of cancer drugs. Figure S3: The interaction of REEP6 and its associated proteins was analyzed using STRING database (https://string-db.org/ (accessed on 30 April 2021)).
