# Supplementary material for: The Clinical and Biological Effects of Receptor Expression-Enhancing Protein 6 in Tongue Squamous Cell Carcinoma
Source: Biomedicines. 2023 Apr 25;11(5):1270. doi: 10.3390/biomedicines11051270 (PMC10215315; doi:10.3390/biomedicines11051270)
Supplement: Supplementary file 1 [file biomedicines-11-01270-s001.zip › biomedicines-2331681-supplementary.pdf]

Figure S1. The full-length blot.

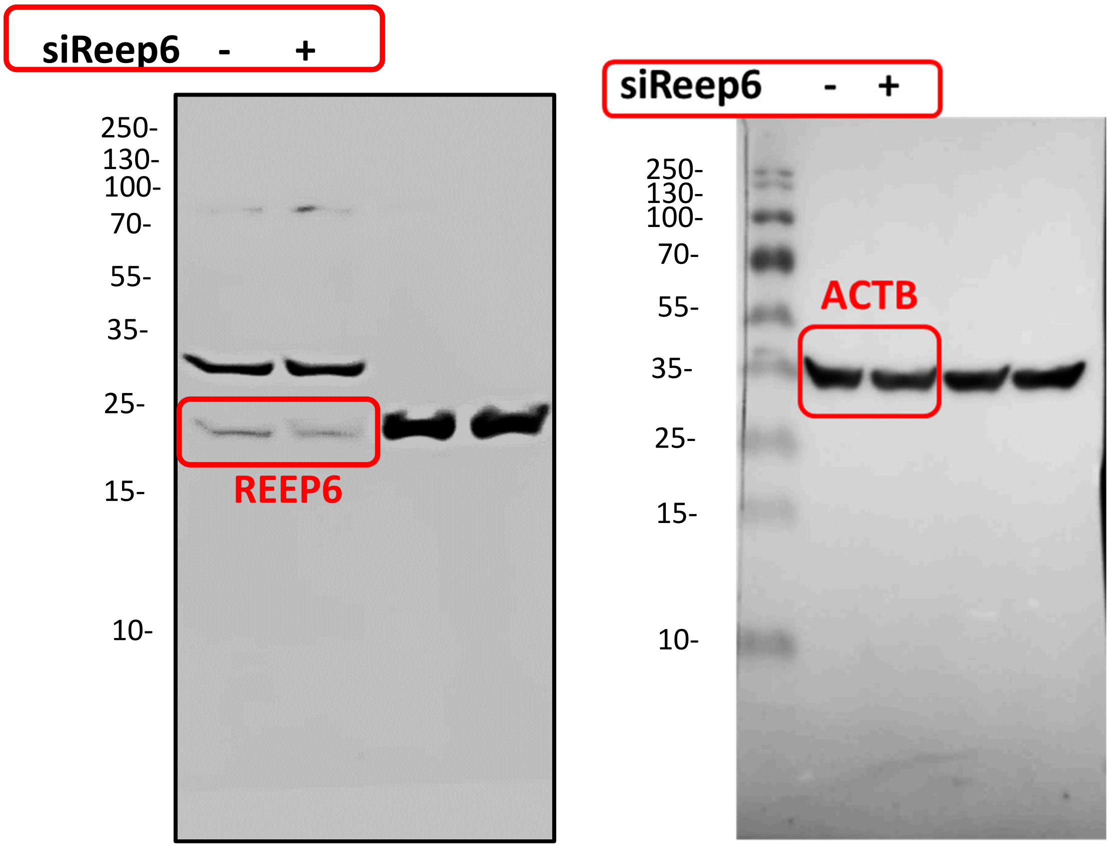

Figure S2. The cell viability of REEP6-silenced SAS monolayer cultures in the presence of cancer drugs. The cell viability of SAS monolayer cultures silenced with scrambled siRNA or siRNA against REEP6 for 3 days was detected in the absence or presence of cisplatin (CIS, 100 µM) or paclitaxel (PTX, 0.2 µM) for 24 h. The 10 nM scrambled siRNA (siCtrl) or siRNA against REEP6 (siREEP6) were transfected into SAS cells for 72 h. All data were represented as the average ± SD from 3 independent experiments. The significant differences between scramble control and knock-downed cells were indicated as *p<0.05 and **p<0.01.

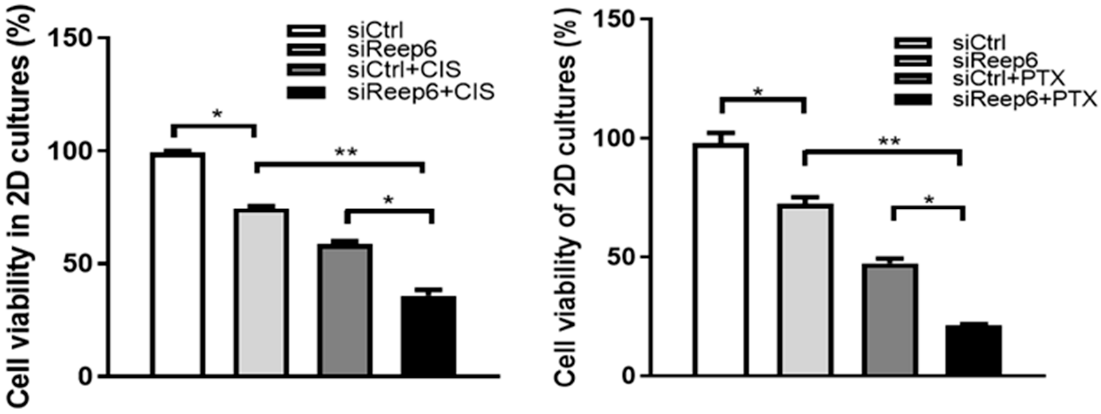

Figure S3. The interaction of REEP6 and its associated proteins was analyzed using STRING database (https://string-db.org/). (RTN1-2: Reticulon 1-2; ATL1-4: Atlastin GTPase 1-4; TMEM33: Transmembrane Protein 33 ; KLRF1: Killer cell lectin-like receptor subfamily F member 1; SNX15: Sorting nexin 15; NXNL2: Nucleoredoxin like 2)

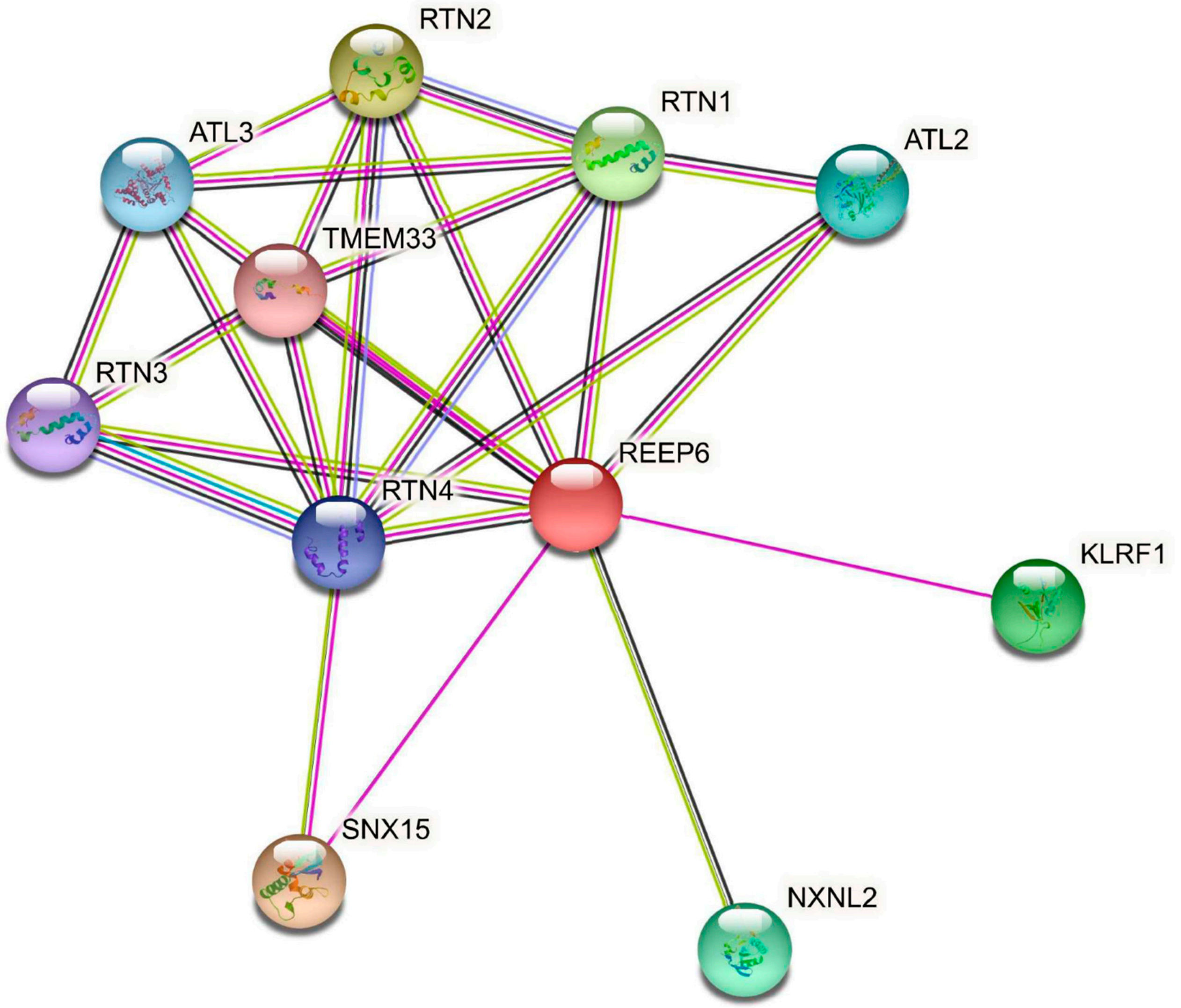

Table S1. The association of REEP6 expression with overall survival of oral cancer patients in TCGA database

Variable	Reep6	No. (%)	CHR (95% CI)	*p value**	AHR (95% CI)	*p value*†
Sex						
Female	Low	46 (56.1)	1.00		1.00	
	High	36 (43.9)	1.08 (0.55−2.13)	0.829	0.99 (0.49−1.99)	0.979[a]
Male	Low	101 (55.5)	1.00		1.00	
	High	81 (44.5)	1.27 (0.81−1.97)	0.299	1.39 (0.89−2.18)	0.152[a]
Age, yrs						
≦60	Low	60 (54.1)	1.00		1.00	
	High	51 (45.9)	1.11 (0.62−2.00)	0.722	1.27 (0.70−2.31)	0.442[a]
＞60	Low	87 (56.9)	1.00		1.00	
	High	66 (43.1)	1.28(0.79−2.06)	0.319	1.26 (0.78−2.06)	0.346[a]
Cell differentiation						
Well	Low	19 (50.0)	1.00		1.00	
	High	19 (50.0)	1.00 (0.31−3.26)	1.000	1.14 (0.35−3.70)	0.829[b]
Moderate, poor	Low	128 (56.6)	1.00		1.00	
	High	98 (43.4)	1.29 (0.87−1.91)	0.206	1.28 (0.87−1.90)	0.216[b]
AJCC pathological stage						
I, II	Low	34 (59.6)	1.00		1.00	
	High	23 (40.4)	1.01 (0.30−3.42)	0.983	1.05 (0.31−3.58)	0.945[c]
III, IV	Low	113 (54.6)	1.00		1.00	
	High	94 (45.4)	1.30 (0.88−1.92)	0.191	1.33 (0.90−1.97)	0.152[c]
T classification						
T1, T2	Low	66 (63.5)	1.00		1.00	
	High	38 (36.5)	0.68 (0.29−1.58)	0.365	0.74 (0.32−1.74)	0.495[d]
T3, T4	Low	81 (50.6)	1.00		1.00	
	High	79 (49.4)	1.40 (0.92−2.13)	0.119	1.42 (0.93−2.16)	0.108[d]
N classification						
N0	Low	63 (53.4)	1.00		1.00	
	High	55 (46.6)	1.66 (0.87−3.19)	0.127	1.79 (0.91−3.52)	0.089[e]
N1, N2	Low	84 (57.5)	1.00		1.00	
	High	62 (42.5)	1.07 (0.67−1.70)	0.775	0.98 (0.61−1.55)	0.917[e]
Postoperative RT						
No	Low	57 (57.6)	1.00		1.00	
	High	42 (42.4)	1.15 (0.65−2.07)	0.629	1.05 (0.58−1.90)	0.880[a]
Yes	Low	77 (55.4)	1.00		1.00	
	High	62 (44.6)	1.54 (0.87−2.72)	0.137	1.59 (0.90−2.81)	0.111[a]

AJCC: American Joint Committee on Cancer, RT: Radiotherapy, CHR: Crude Hazard Ratio, AHR: Adjusted Hazard Ratio, CI: Confidence Interval.
p values: measured by univariate Cox's regression; p values: p values †: measured by multivariate Cox's regression*
p values [a]: adjusted for cell differentiation (well vs. moderate, poor) and AJCC pathological stage (I, II vs. III, IV)
p values [b]: adjusted for AJCC pathological stage (I, II vs. III, IV)
p values [c]: adjusted for cell differentiation (moderate, poor vs. well)
p values [d]: adjusted for cell differentiation (well vs. moderate, poor) and N classification (N0 vs. N1, N2)
p values [e]: adjusted for cell differentiation (well vs. moderate, poor) and T classification (T1, T2 vs. T3, T4)

Table S2. The co-expression of REEP6/EMT markers in DFS of oral cancer patients in TCGA database

Variable		No. (%)	CHR (95% CI)	*p value**	AHR (95% CI)	*p value* †
REEP6	Low	134 (59.3)	1.00		1.00	
	High	92 (40.7)	1.56 (0.92−2.64)	0.101	1.60 (0.94−2.71)	0.085[a]
E-Cad	Low	15 (6.6)	1.00		1.00	
	High	211 (93.4)	4.13 (0.57−29.85)	0.160	4.30 (0.60−31.12)	0.148[a]
N-Cad	Low	211 (93.4)	1.00		1.00	
	High	15 (6.6)	1.74 (0.75−4.07)	0.200	1.65 (0.70−3.88)	0.251[a]
VIM	Low	196 (86.7)	1.00		1.00	
	High	30 (13.3)	1.48 (0.75−2.94)	0.261	1.30 (0.65−2.60)	0.455[a]
SNAIL	Low	164 (72.6)	1.00		1.00	
	High	62 (27.4)	1.29 (0.73−2.29)	0.384	1.21 (0.68−2.16)	0.509[a]
TWIST1	Low	218 (96.5)	1.00		1.00	
	High	8 (3.5)	2.29 (0.91−5.76)	0.079	2.55 (1.01−6.45)	0.047[a]
REEP6 (L), E-Cad (H)		9 (4.0)	1.00		1.00	
REEP6 (L), E-Cad (L)		6 (2.7)	0.70 (0.10−5.09)	0.728	6040.45 (0.00−3.51 $\times 10^{53}$)	0.882[b]
REEP6 (H), E-Cad (L)		125 (55.3)	0.77 (0.46−1.31)	0.343	7544.43 (0.00−4.32 $\times 10^{53}$)	0.879[b]
REEP6 (H), E-Cad (H)		86 (38.1)	1.61 (0.95−2.74)	0.077	11181.48 (0.00−6.40 $\times 10^{53}$)	0.873[b]
REEP6 (L), N-Cad (L)		120 (53.1)	1.00		1.00	
REEP6 (H), N-Cad (L)		91 (40.3)	1.59 (0.94−2.70)	0.084	1.83 (1.04−3.22)	0.037[b]
REEP6 (L), N-Cad (H)		14 (6.2)	1.92 (0.82−4.48)	0.133	2.57 (1.04−6.38)	0.042[b]
REEP6 (H), N-Cad (H)		1 (0.4)	0.05 (0.00−3.90 $\times 10^{5}$)	0.710	0.00 (0.00−5.56 $\times 10^{231}$)	0.972[b]
REEP6 (L), VIM(L)		110 (48.7)	1.00		1.00	
REEP6 (H), VIM(L)		86 (38.1)	1.65 (0.97−2.81)	0.063	2.04 (1.12−3.71)	0.019[b]
REEP6 (L), VIM(H)		24 (10.6)	1.78 (0.87−3.64)	0.115	2.53 (1.13−5.62)	0.023[b]
REEP6 (H), VIM(H)		6 (2.7)	0.57 (0.08−4.14)	0.581	0.89 (0.12−6.64)	0.906[b]
REEP6 (L), SNAIL (L)		94 (41.6)	1.00		1.00	
REEP6 (H), SNAIL (L)		70 (31.0)	1.37 (0.79−2.35)	0.263	1.71 (0.90−3.24)	0.101[b]
REEP6 (L), SNAIL (H)		40 (17.7)	1.09 (0.55−2.17)	0.804	1.50 (0.69−3.27)	0.311[b]
REEP6 (H), SNAIL (H)		22 (9.7)	1.51 (0.68−3.33)	0.312	2.01 (0.83−4.85)	0.120[b]
REEP6 (L), TWIST1(L)		129 (57.1)	1.00		1.00	
REEP6 (H), TWIST1(L)		89 (39.4)	1.43 (0.84−2.42)	0.190	1.61 (0.92−2.80)	0.057[b]
REEP6 (L), TWIST1(H)		5 (2.2)	1.98 (0.62−6.34)	0.252	2.53 (0.76−8.41)	0.131[b]
REEP6 (H), TWIST1(H)		3 (1.3)	2.74 (0.66−11.36)	0.165	3.53 (0.83−15.07)	0.089[b]

DFS: Disease-Free Survival; H: High expression, L: Low expression, CHR: Crude Hazard Ratio, AHR: Adjusted Hazard Ratio, CI: Confidence Interval; E-Cad: E-cadherin; N-Cad: N-cadherin; VIM: Vimentin.

p values: measured by univariate Cox's regression; p values †: measured by multivariate Cox's regression.*

p values[a] : adjusted for cell differentiation (well vs. moderate, poor) and AJCC pathological stage (I, II vs. III, IV) by multivariate Cox's regression.

p values[b]: adjusted for group comparison by multivariate Cox's regression.

Table S3. The comparison of REEP6 expression between normal, tumor adjacent normal and tumor tissues of TSCC patients

Variables	Normal tissue		Tumor adjacent normal		Tumor		χ^2	*p*-value*
	Mean±SD	Median	Mean±SD	Median	Mean±SD	Median		
	(n=35)		(n=201)		(n=250)			
REEP6	0.14 ± 0.69[ab]	0.00	0.47 ± 0.94[a]	0.00	0.45 ± 0.93[b]	0.00	5.987	0.050

Abbreviations: SCC, squamous cell carcinoma; SD, standard deviation.

**p values: estimated by Kruskal-Wallis one-way ANOVA test.*

[a]p=0.028; [b]p=0.012.

χ^2: Chi-squared test.

Table S4. The association of REEP6 expression with clinicopathologic outcomes in TSCC patients

Variable	No. (%)	Mean±SD	Median	*p*-value
Sex				
Female	31 (12.4)	0.45 ± 0.81	0.00	0.982*
Male	219 (87.6)	0.45 ± 0.94	0.00	
Age, y				
≦50	130 (52.0)	0.42 ± 0.91	0.00	0.659*
＞50	120 (48.0)	0.48 ± 0.95	0.00	
Cell differentiation				
Well	27 (10.8)	0.44 ± 0.89	0.00	
Moderate	206 (82.4)	0.43 ± 0.86	0.00	0.657†
Poor	17 (6.8)	0.65 ± 1.58	0.00	
AJCC pathological stage				
I, II	170 (68.0)	0.45 ± 0.88	0.00	0.902*
III, IV	80 (32.0)	0.44 ± 1.02	0.00	
T classification				
T1, T2	197 (78.8)	0.47 ± 0.96	0.00	0.533*
T3, T4	53 (21.2)	0.38 ± 0.81	0.00	
N classification				
N0	198 (79.2)	0.46 ± 0.89	0.00	0.700*
N1, N2	52 (20.8)	0.40 ± 1.07	0.00	

Abbreviations: AJCC, American Joint Committee on Cancer.

* *p values: estimated by student's t-test;* † *p values: estimated by one-way ANOVA test.*
